# Anti-HCV NS2-3 potential of selected plant bioactive compounds revealed by docking, simulation and DFT

**DOI:** 10.1038/s41598-025-18577-8

**Published:** 2026-02-19

**Authors:** Clement I. Mboto, Elizabeth N. Mbim, Uwem O. Edet, Moses Lugos, Mohnad Abdalla, Wilfred O. Ndifon, Eno E. Ebenso, Samuel. I. Udo, Henry O. Egharevba, Uwem E. George, Mohamed H El-Sayed, Sami Fatehi Abdalla

**Affiliations:** 1 Department of Microbiology, Faculty of Biological Science, University of Calabar, Calabar, Cross River State Nigeria; 2 Viro-Bio Research Laboratory, Department of Microbiology, Faculty of Biological Science, University of Calabar, Calabar, Cross River State Nigeria; 3 Department of Microbiology, Faculty of Sciences, Cross State University of Technology, Calabar, Cross River State Nigeria; 4 Department of Biological (Microbiology), Faculty of Natural and Applied Sciences, Arthur Jarvis University, Akpabuyo, Cross River State Nigeria; 5 Department of Microbiology, Faculty of Science, University of Jos, Jos, Pleateau State Nigeria; 6 Pediatric Research Institute, Children’s Hospital Affiliated to Shandong University, Jinan, 250022 China; 7 Department of Community Medicine, University of Calabar, Calabar, Nigeria; 8 School of Mathematical and Physical Sciences, Faculty of Agriculture, Science & Technology North West University (Mafikeng Campus), Mmabatho, 2735 South Africa; 9 National Institute for Pharmaceutical Research and Development (NIPRD), Idu Industrial Layout, Idu, Garki, Abuja Nigeria; 10 Redeemer’s University, off Ibadan-Oshogbo Road, Ede, Osun State Nigeria; 11 Department of Biological Sciences, College of Sciences, Northern Border University, Arar, Saudi Arabia; 12 Department of Clinical Sciences, College of Medicine, University of Almaarefa, Daryiyah, 13713 Saudi Arabia; 13 Research Center, Deanship of Scientific Research and Post-Graduate Studies, AlMaarefa University, Daryiyah, 13713 Riyadh Saudi Arabia

**Keywords:** HCV, Bioactive compounds, DFT, Molecular docking, *Jatropha tanjorensis* and *Solanum nigrum*, Simulation, Computational biology and bioinformatics, Drug discovery, Microbiology

## Abstract

Presently, there is no vaccine for hepatitis C virus (HCV) and available drugs present with adverse effects that have prompted the search for newer and safer alternatives. The present study evaluated the anti-HCV potential of selected bioactive compounds from *Jatropha tanjorensis* and *Solanum nigrum* against HCV non-structural (NS2-3) protein. The selected bioactive compounds (3-methoxy-4-methylaniline, 2,2’-Azoxybis[3-methylpyridine], isopropyl thiophosphondiamide, and squalene) were screened for compliance with Lipinski’s role five (LRF) and toxicity using the MCULE tool. Furthermore, the ligands were docked against the NS2-3 (2hd0) protein with ledipasvir, and a co-crystal as controls using the Autodock Vina tool. Docking scores were generated using the London dG scoring function. Following docking, a 200 nanosecond (nsec) simulation run was performed using the Schrodinger Desmond module. In addition, density functional theory (DFT) was utilised to evaluate their reactivities. The selected compounds were not toxic and obeyed the LRF. Molecular docking scores for ledipasvir and the co-crystal were − 8.8 and − 6.3 kcal/mol, respectively while of the ligands ranged from − 3.5 to -7.3 kcal/mol, implying favourable bindings. The amino acid residues involved in the binding were those within the active site of the target protein. RMSD values indicated that isopropyl thiophosphondiamide was the most stable ligand. PSA, MolSA and SASA values suggest stability and availability for water contact. DFT calculations indicate that the compounds were moderately stable and highly reactive, with energy gaps that ranged from 0.5810 to 1.0621 eV. The favourable pharmacokinetics and docking outputs observed in this study needs to be further validated using in vitro and in vivo studies.

## Introduction

Globally, infection with hepatitis C virus (HCV) is one of the leading causes of liver diseases and hepatocellular carcinoma, with an estimated 71.1 million individuals chronically infected worldwide^1^. The HCV genome is single-stranded, positive-sense RNA of approximately 9.6 kb in length. Its genome is highly structured, comprising a single open reading frame (ORF) that encodes a polypeptide that is processed by cellular and viral proteases to generate 10-polypeptides. The HCV genome also contains an overlapping reading frame that may lead to the synthesis of an additional protein^1,2^. The virus exhibits a high degree of genetic heterogeneity, and it has been classified into seven major genotypes (HCV 1–7), including 67 confirmed and 20 provisional subtypes, on the basis of phylogenetic analysis of its genome sequences^3^. There is also a significant variation in its circulating genotypes across the globe^3,4^. HCV genotype 1 has a global prevalence of 49.1%, followed by genotypes 3 (17.9%), 4 (16.8%), and 2 (11.0%) and continues to spread globally^3^.

Vertical transmission and contacts with contaminated body fluids, tissues, and equipment constitute its main transmission routes^5^. In many developing countries where intravenous drug abuse and male-to-male sex are not too common, transmission of HCV is predominantly through the transfusion of unscreened blood and unprotected sex^5,6^. Increasing incidences of infection with the virus have become a global norm since it was first isolated in 1989^6,7^. With no HCV vaccine, the management of its infection is presently limited to the use of antiviral drugs, with most targeting non-structural proteins (NS) (proteases) essential for viral replication^8,9^. The HCV NS2-3 protein complex in HCV has pivotal roles in the virus’s life cycle. It is involved in HCV polyprotein processing, RNA replication, and viral particle assembly^10^. Studies have revealed that during proteolytic processing, NS3, a serine protease, along with the NS4A co-factor, cleaves the HCV polyprotein at the NS2-3 junction, generating mature viral proteins^11,12^. Similarly, the NS2 component of the complex regulates NS3 protease activity, ensuring precise cleavage and functional protein production^13^. Furthermore, both NS2 and NS3 interact with intracellular membranes, including the endoplasmic reticulum, enhancing their roles in viral replication and assembly. As an RNA helicase, NS3 unwinds RNA during replication, while the entire NS2-3 complex contributes to the assembly and release of infectious viral particles, facilitating the formation of its replication complex and enhancing viral pathogenesis^13,14^. Thus, its importance in the HCV life cycle makes it an potent drug target^15–18^.

Despite the availability of HCV drugs, majority of them are only effective in approximately 50% of infected individuals, with most associated with undesirable side effects, toxicities, complications, and are being incapable of preventing re-infection^14^. These challenges have prompted the search for newer, better and safer antiviral agents. Put together, the management of HCV in the absence of a vaccine and safer drugs that do not present with side effects poses a significant global public health problem^19^. Furthermore, available drugs such as the pan-genotypic direct-acting antivirals (DAAs) for adults with chronic hepatitis C comes with a number of issues. First, many countries especially those in resource poor settings possess limited health infrastructures^19^. Other issues include their lack of specificity and stability, unpleasant side effects, toxicities, formation of escape mutants, and poor accessibility^19^.

Thus, there is a heightened search for newer, more potent, and safer alternatives targeting the various viral proteins^14,19,20^. Several studies have revealed that ethno-medicinal plants possess potent bioactive compounds with excellent properties that could be employed in the management of several ailments, including HCV^21–26^. The present aimed to evaluate using in silico approaches (toxicity profiling, LRF, DFT, molecular docking and simulation), the potential of selected compound from two medicinal plants (*Jatropha tanjorensis* and *Solanum nigrum*) reportedly utilised to manage HCV locally and other viral hepatitis in Nigeria. In addition, the study also sought to understand the potential mode of action of these selected compounds.

## Methods

### Retrieval of ligands and target protein

The ligands were recovered from an earlier study that profiled a total of 35 bioactive compounds recovered from *Jatropha tanjorensis* (*n* = 7) and *Solanum nigrum* berries (*n* = 28) following GC-MS^4^. From each of the bioactive compounds, two bioactive compounds were selected based on their peak concentrations (highest peaks). The selected bioactive compounds, which included squalene and 3-Methoxy-4-methylaniline from *J. tanjorensis leaves*, and 2,2’-Azoxybis[3-methylpyridine], and isopropyl thiophosphondiamide from *S. nigrum*. In addition to their favourable chromatogram results, the compounds were further selected based on their chemical diversity in terms of their functional groups and their potential to bind to the NS2-3 protease active site to allow for the exploration of a wide range of binding interactions such as hydrophobic, hydrogen bonding, and covalent bonds. On the other hand, the 3-dimensional structure of the NS2-3 protein, was retrieved from the Research Collaboratory for Structural Bioinformatics (RCSC) (http://www.rcsb.org/pdb/home/home.do) and the protein database (pdb). Details of the retrieved proteins, such as the source, name of the protein, PDB ID, Uniprot name, Uniprot accession ID, Uniprot taxonomic ID, and organism were sc-PDB, Protease NS2-3, 2hd0, POLG_HCVH, P27958, 11,108, and Hepatitis C virus genotype 1a, respectively.

### Toxicity/biological profiling of selected bioactive compounds

The SMILES strings of the selected bioactive compounds were obtained using PUBCHEM and utilised in the prediction of the toxicity and biological properties of the ligands. This was done as previously reported^27,28^. All four ligands showed no potential toxic functional groups and were utilised for analysis.

### Molecular docking analysis

#### Ligands and target protein Preparation

The selected ligands and the target protein were prepared for docking analysis as reported^29–31^. First, using ChemDraw software, the structures of the ligands were sketched, their energies minimised and the resulting stable structures saved in MDL SD format^29,32^. The utilised target protein, a NS2-3 protease (2hd0) was prepared as reported earlier^31^. In preparing the protein, the native ligand was revealed, the docking coordinates defined, incomplete charges removed and the resulting protein saved for docking^31^. The resolution of the protein was 2.280, and the default binding sites of the NS2-3 protein (2hd0) were X ( 95.9848), Y (22.0656) and Z (57.1135).

#### Molecular docking (MD) and simulation (MDS)

The molecular docking was performed as reported earlier using the allosite tool hosted at https://mdl.shsmu.edu.cn/AST/^30,31^. In performing the docking, the active site was identified in the allosteric tool, and this was followed by the MD using the aid of the Autodock Vina tool and the binding scores for the various ligands as they interacted with the target protein were generated^30,31^. Docking outputs were visualised in two and three dimensions using Pymol^33^ and Biovia Discovery Studio. Following MD, a 200 nsec MDS was carried for the various complexes formed and this was done using the Schrodinger Desmond module. To ensure the presence of water in the MDS environment, TIP3P model was selected while isosmotic condition was achieved via the addition of 0.15 M NaCl, and the boundary of the boundary of the simulation box as set at 10 Å. Furthermore, the system was allowed to reached equilibrium with temperature and pressure set 310 K and 1.013 bar, respectively^30,31^.

### Density functional theory (DFT)

Geometric structures of the bioactive compounds were optimized using DFT at the ωB97XD, M062X, B3LYP, and HSE with 6-311 + + G (d, p) method of theory using the Gaussian 16 and Gauss View 6.0.16 software^34^. The Highest Occupied and Lowest Unoccupied Molecular Orbitals (HOMO-LUMO) were carried out at B3LYP/6-311 + + G(d, p).

## Results and discussion

### Drug-likeness properties of the selected ligands

Table 1 shows the result of the toxicity and basic profiles of the selected bioactive compounds. The result indicate that the selected compounds were non-toxic. This implies that the selected compounds squalene, 3-Methoxy-4-methylaniline, 2,2’-Azoxybis[3-methylpyridine], and isopropyl thiophosphondiamide will not be toxic to the liver and other cellular organs in vivo^35^. The selected compounds did not violate the LROF as they had 0 to 1 violations only. As posited by Lipinski et al.^36^ and Pires et al.^37^, for a compound to be classified as a potential drug, it must obey the LRF, one of the most utilised approaches in the initial stages of drug development and improvement^35,37^.

**Table 1:** Toxicity/basic properties of selected non-toxic bioactive compounds.

Properties	A	B	C	D
Toxicity profiling	None	None	None	None
Mass	328.4872	396.6902	396.6471	137.1788
LogP	6.5489	8.8300	7.3308	2.1670
H-bond acceptors	2	0	1	2
H-bond donors	1	0	1	1
Rotatable bonds	14	6	4	1
PSA	37.3000	0	20.2300	35.2500
RO5 violations	1	1	1	0
RO3 violations	3	3	3	0
Refractivity	106.7958	131.5930	127.4738	42.3044
Atoms	56	77	73	21
Rings	0	4	4	1
Heavy atoms	24	29	29	10
Hydrogen atoms	32	48	44	11
Heteroatoms	2	0	1	2
N/O atoms	2	0	1	2
Inorganic atoms	0	0	0	0
Halogen atoms	0	0	0	0
Chiral centers	0	8	8	0
R/S chiral centers	0	8	8	0
Unknown chiral centers	0	0	0	0
Undefined chiral centers	0	0	0	0
Stereo double bonds	6	0	1	0
Cis/trans stereo double bonds	0	0	0	0
Unknown stereo double bonds	6	0	1	0
Undefined stereo double bonds	0	0	0	0

Key: a = Squalene; b = 2,2’-Azoxybis[3-methylpyridine]; c = Isopropylthiophosphondiamide; d = 3-Methoxy-4-methylaniline.

### HOMO-LUMO properties of the ligands

**Fig. 1:**
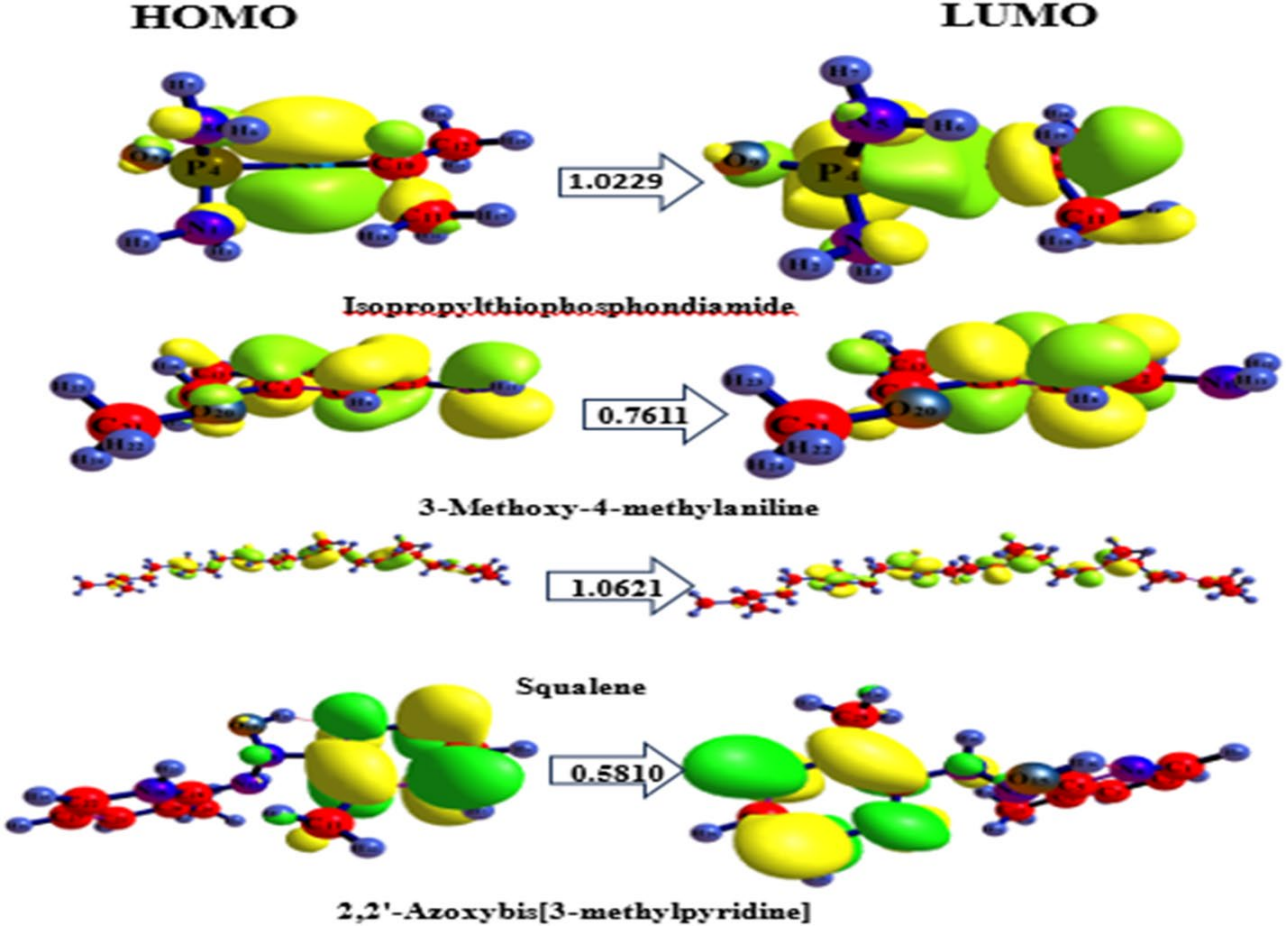
HOMO-LUMO and energy gaps calculated for the study compounds.

In addition to the LRF and toxicity profiling of the ligands, the highest occupied molecular orbitals (HOMO) and the lowest unoccupied molecular orbitals (LUMO) of the bioactive compounds were also evaluated. The HOMO depicts the ability of a compound to donate electrons within the molecule, the LUMO reveals the capacity of a compound to accept electrons^38,39^. Collectively called quantum descriptor, these parameters have become relevant in drug discovery as they describe chemical stability and reactivity^24^. These parameter include chemical hardness (η), ionization potential (IP), electronegativity (χ), chemical softness (σ), electrophilicity index (ω), EA and chemical potential (µ) of the studied complex and are reported in Table 2; Fig. 1, respectively. Their energy gaps ranged from 0.5810 to 1.0621 eV. Specifically, squalene and isopropyl thiophosphondiamide had higher energy gaps of 1.0229 and 1.0621 eV compared to other compounds (2,2’-azoxybis [3-methylpyridine and 3-methoxy-4-methylaniline that ranged from 0.5810 to 0.7611 eV, respectively. As noted by Zara et al.^40^ low energy gap values emanate from loose electrons readily available in a system, enhancing the rapid electronic transition and thus, informing the reactivity of the molecules.

The ionization potential (IP) values which indicate the energies required to remove electrons from the HOMO was least (4.2074 eV) in 2,2’-azoxybis[3-methylpyridine] and highest (8.1716 eV) in 3-methoxy-4-methylaniline. As stated by Rizwana et al.^41^, the measure of the biological activity of the study compounds is evaluated by their ionisation potential and electron affinity, among other indicators. 3-Methoxy-4-methylaniline had the highest chemical hardness (4.0858 eV), while 2,2’-azoxybis[3-methylpyridine] presented the highest chemical softness (0.2377 eV). According to Benjamin et al.^42^ the electrophilicity index (ω) is a measure of the affinity for electrons, and during the evaluation of a molecule’s biological activity, the electrophilicity index confirms the pathway for the molecular interactions between the study molecules and proteins of interest, and also aligns with previous reports^43–45^. Our compounds showed electrophilicity index (ω) that ranged from 1.7130 to 2.9264 eV (Table 2).

**Table 2:** HOMO-LUMO energies and their corresponding quantum chemical parameters.

System	HOMO (eV)	LUMO (eV)	IP (eV)	EA (eV)	Eg (eV)	η (eV)	σ (eV)	χ (eV)	µ (eV)	ω (eV)
A	− 7.3844	− 1.0621	6.3223	7.3844	1.0621	3.1612	0.1582	4.2232	− 4.2232	2.8211
B	− 8.9326	− 0.7611	8.1716	8.9327	0.7611	4.0858	0.1224	4.8469	− 4.8469	2.8749
C	− 4.7884	− 0.5810	4.2074	4.7884	0.5810	2.1037	0.2377	2.6847	− 2.6847	1.7130
D	− 8.0407	− 1.0229	7.0178	8.0407	1.0229	3.5089	0.1425	4.5318	− 4.5318	2.9264

Key: a = Squalene; b = 2,2’-Azoxybis[3-methylpyridine]; c = Isopropyl thiophosphondiamide; d = 3-Methoxy-4-methylaniline.

### Molecular Docking analysis

Molecular docking, as demonstrated by Issa et al.^46^, is a technique employed to predict the binding of a ligand to a target, forming a stable complex and thereby determining the optimal spatial arrangement of its molecules. They also emphasized that comprehending this preferred alignment is essential for assessing the strength of the interaction, typically through scoring functions. Studies have further highlighted the significance of amino acid residues engaged in conventional hydrogen bond interactions for understanding various biochemical activities and biological processes^42^.

**Fig. 2:**
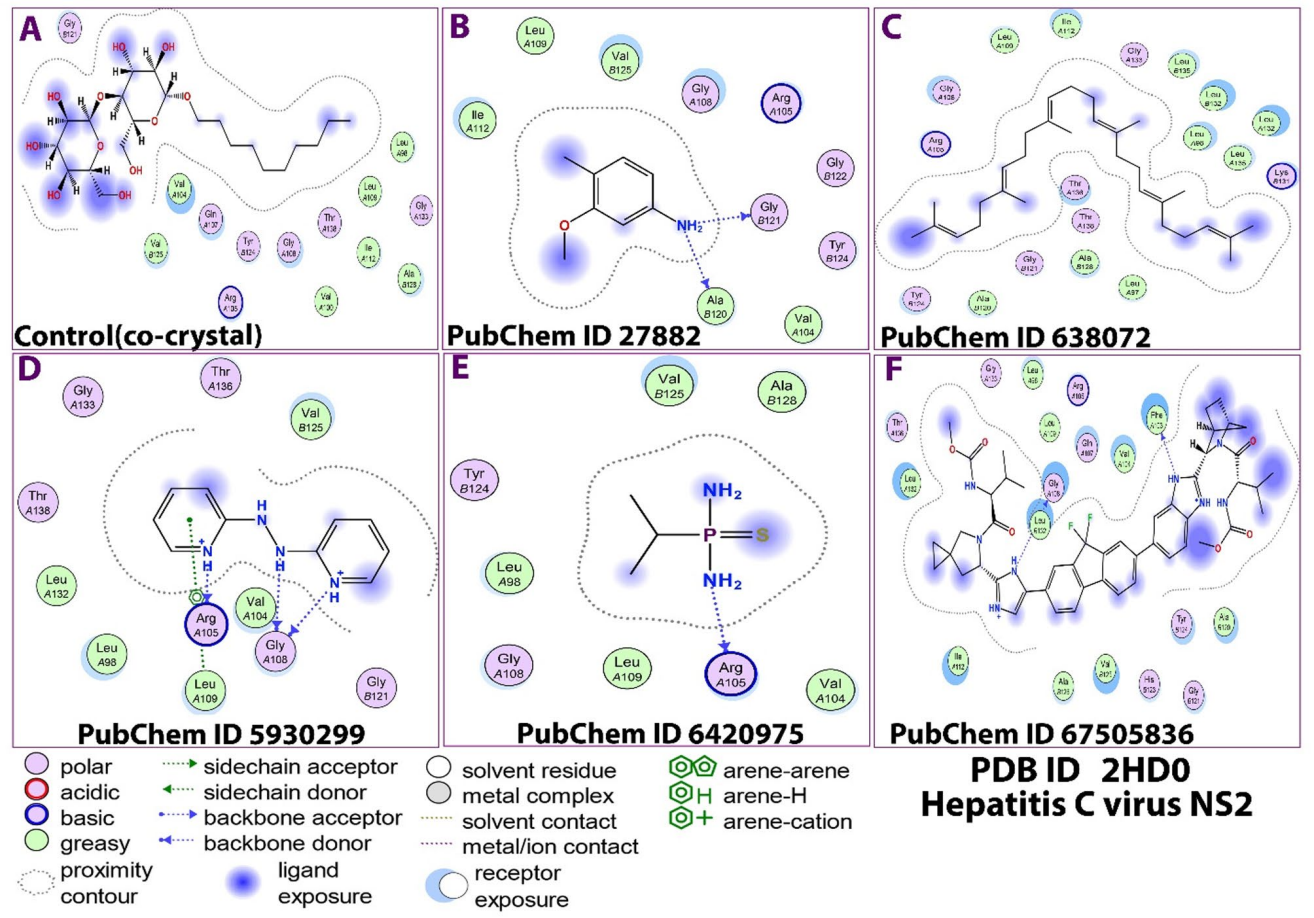
2D binding poses of the co-crystal and the ligands to target protein (2HD0). Key: PubChem CID 27,882 (3-Methoxy-4-methylaniline); PubChem CID 638,072 (squalene); PubChem CID 5,930,299 (2,2’-Azopyridine); PubChem CID 6,420,975 (Isopropyl thiophosphondiamide) and PubChem CID 67,505,836 (Ledipasvir).

**Fig. 3:**
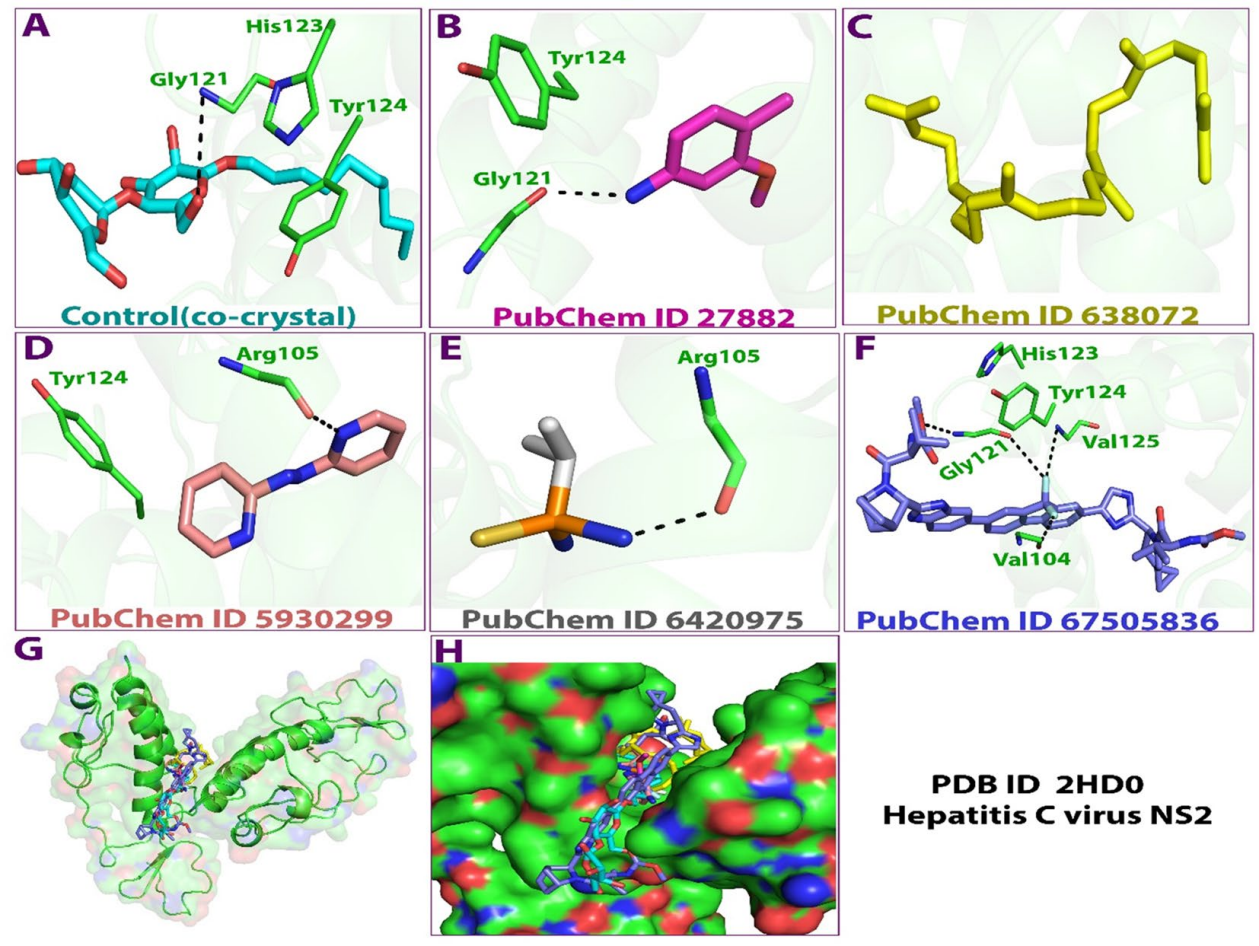
3D binding poses of the ligands and co-crystal with the target protein (2HD0). Key: PubChem CID 27882 (3-Methoxy-4-methylaniline); PubChem CID 638072 (Squalene); PubChem CID 5930299 (2,2’-Azopyridine); PubChem CID 6420975 (Isopropyl thiophosphondiamide) and PubChem CID 67505836 (Ledipasvir).

Figures 2 and 3, and Table 3 show the molecular docking results between the ligands and the protein. Figure 2 shows the binding interaction between the target protein and the various ligands employed in the study. Figure 2A denote binding pose for the control crystal while 2B-IF represent binding poses for the ligands -Methoxy-4-methylaniline, squalene, 2,2’-Azopyridine, Isopropyl thiophosphondiamide and ledipasvir, respectively. Similarly, among the reacting molecules, the most common types of interactions were polar, greasy and basic. In addition, in the interaction between the ledipasvir with the protein, acidic bonds were formed. Furthermore, various interacting amino acids were 14 (Control co-crystal), 10 (3-Methoxy-4-methylaniline)), 18 (squalene and ledipasvir), 11 (2,2’-Azopyridine ) and 8 (Isopropyl thiophosphondiamide). In all these poses, the dominant bond type was hydrogen. Hydrogen is an important bond that is involved in numerous cellular activities^47^. It has been shown to also mediate protein-ligand binding affinity^48^. This implies that all the ligands are capable of interacting strongly with the target protein.

**Table 3:** Binding energies of the ligands and control co-crystal.

Ligands	Binding energy (kcal/mol)
2hd0_Control(co-crystal)	− 6.3
27,882	− 4.6
638,072	− 7.3
5,930,299	− 6.3
6,420,975	− 3.5
67,505,836	− 8.8

Key: PubChem CID 27882 (3-Methoxy-4-methylaniline); PubChem CID 638072 (squalene); PubChem CID 5930299 (2,2’-Azopyridine); PubChem CID 6420975 (Isopropyl thiophosphondiamide) and PubChem CID 67505836 (Ledipasvir).

Figure 3 shows the binding poses of the studied ligands and target protein in 3D. The figure revealed the presence of different bonds and amino acids involved in the interactions. In the interaction involving ligand A (Control co-crystal), the amino acids Tyr124, His123 and Gly121 were involved, while in ligand B, Tyr124 and Gly121 were the only amino acids involved. Similarly, for ligand C, no amino acid was observed in the pose. For ligand D, the amino acids were Tyr124 and Arg105 for ligand E. Furthermore, for ligand F (ledipasvir), the amino acids were His123, Tyr124, Val125, Gly121 and Val104. Amino acid Tyr124 was overlapping in the various interactions. The various binding energies of the ligands and the 2HD0 control (co-crystal) is presented in Table 3. Overall, the docking scores indicate that the ligands can bind and interact with the target protein. Interestingly, all the amino acids involved in the docking were those involved in the catalytic site of the protease which have been identified to include amino acids residues 94–217^49^, implying that these ligands are capable of interfering with the activity of the protease by binding to various resides of its catalytic domain of the NS3 protein. Thus, they can function as anti-HCV agents.

### Molecular simulation results

**Fig. 4:**
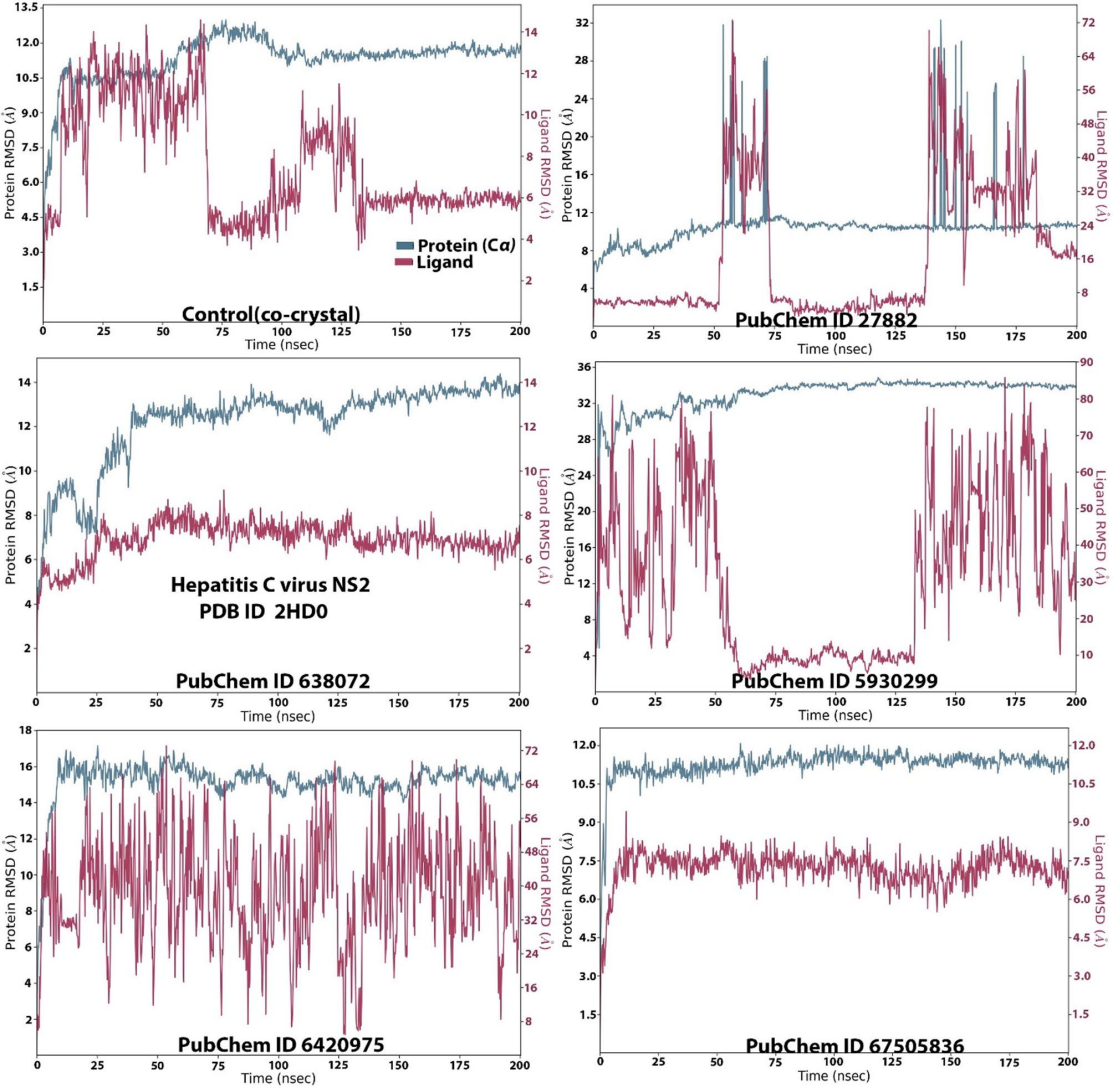
Fluctuations of the RMSD values across the 200 nsec duration of simulation for all the ligands. Key: PubChem CID 27882 (3-Methoxy-4-methylaniline); PubChem CID 638072 (squalene); PubChem CID 5930299 (2,2’-Azopyridine); PubChem CID 6420975 (Isopropyl thiophosphondiamide) and PubChem CID 67505836 (Ledipasvir).

**Fig. 5:**
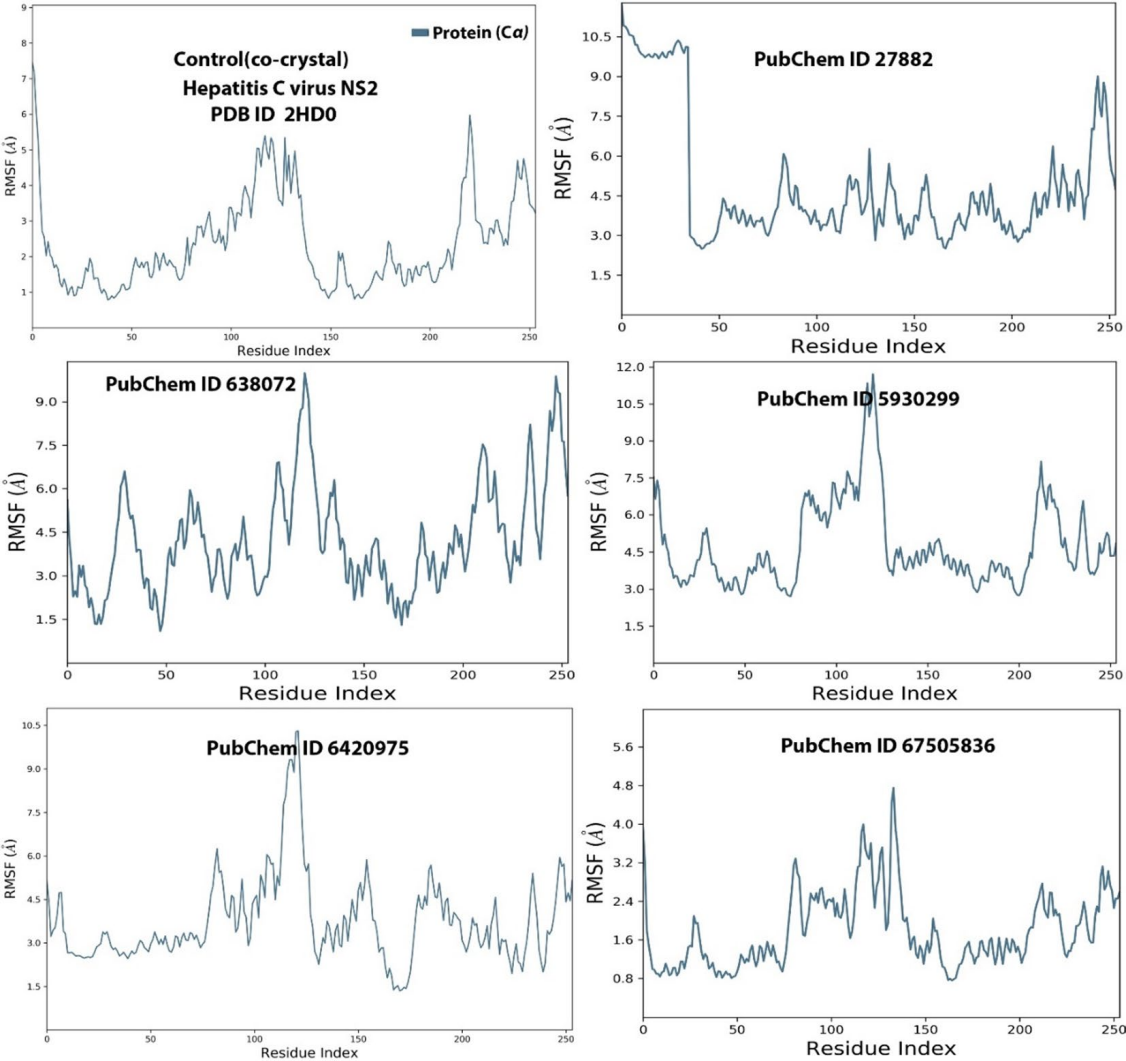
RMSF values of the various ligands and the control co-crystals. Key: PubChem CID 27882 (3-Methoxy-4-methylaniline); PubChem CID 638072 (squalene); PubChem CID 5930299 (2,2’-Azopyridine); PubChem CID 6420975 (Isopropyl thiophosphondiamide) and PubChem CID 67505836 (Ledipasvir).

**Fig. 6:**
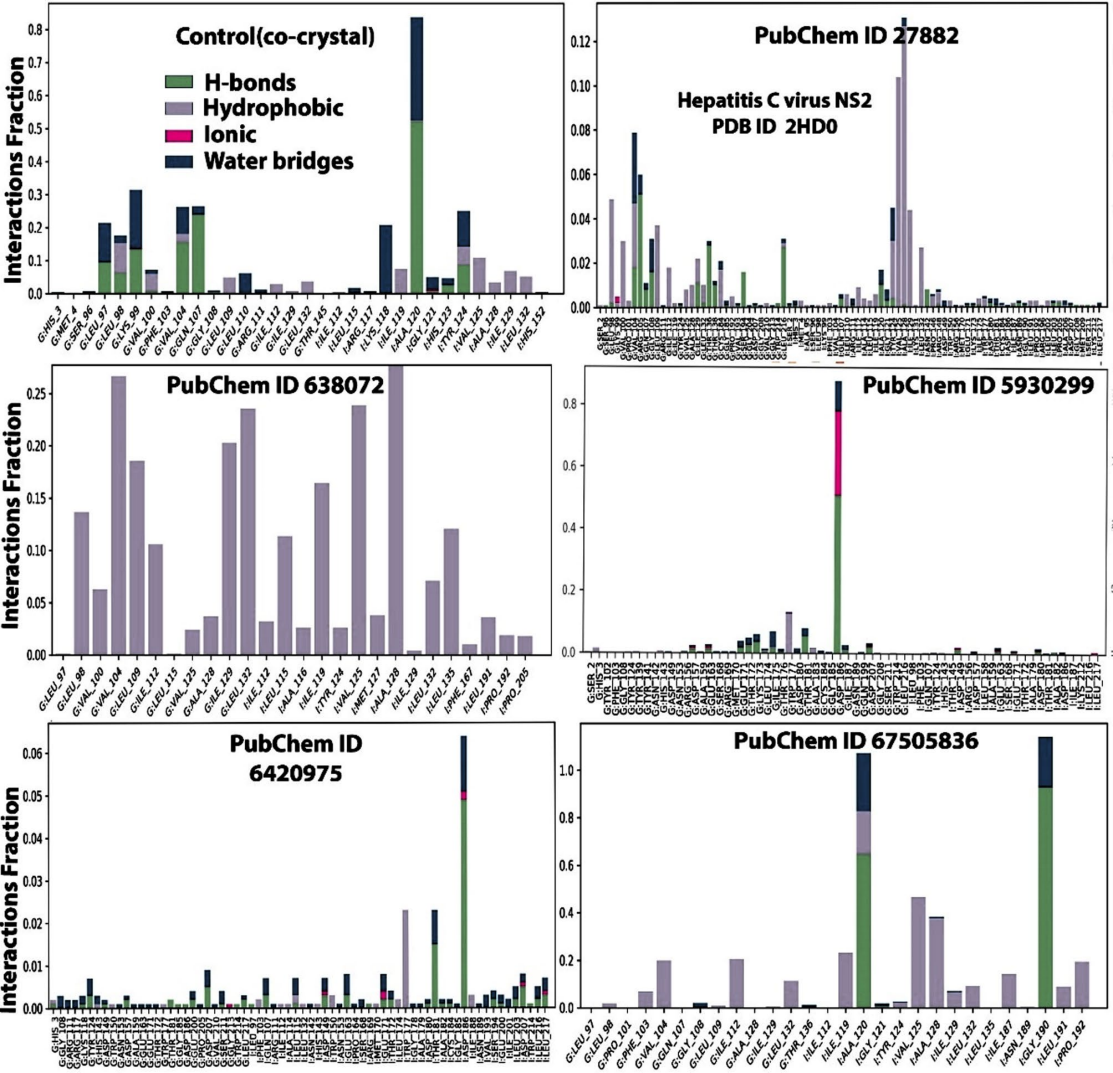
A graphical illustration of the various protein-ligand contacts. Key: PubChem CID 27882 (3-Methoxy-4-methylaniline); PubChem CID 638072 (squalene); PubChem CID 5930299 (2,2’-Azopyridine); PubChem CID 6420975 (Isopropyl thiophosphondiamide) and PubChem CID 67505836 (Ledipasvir).

**Fig. 7:**
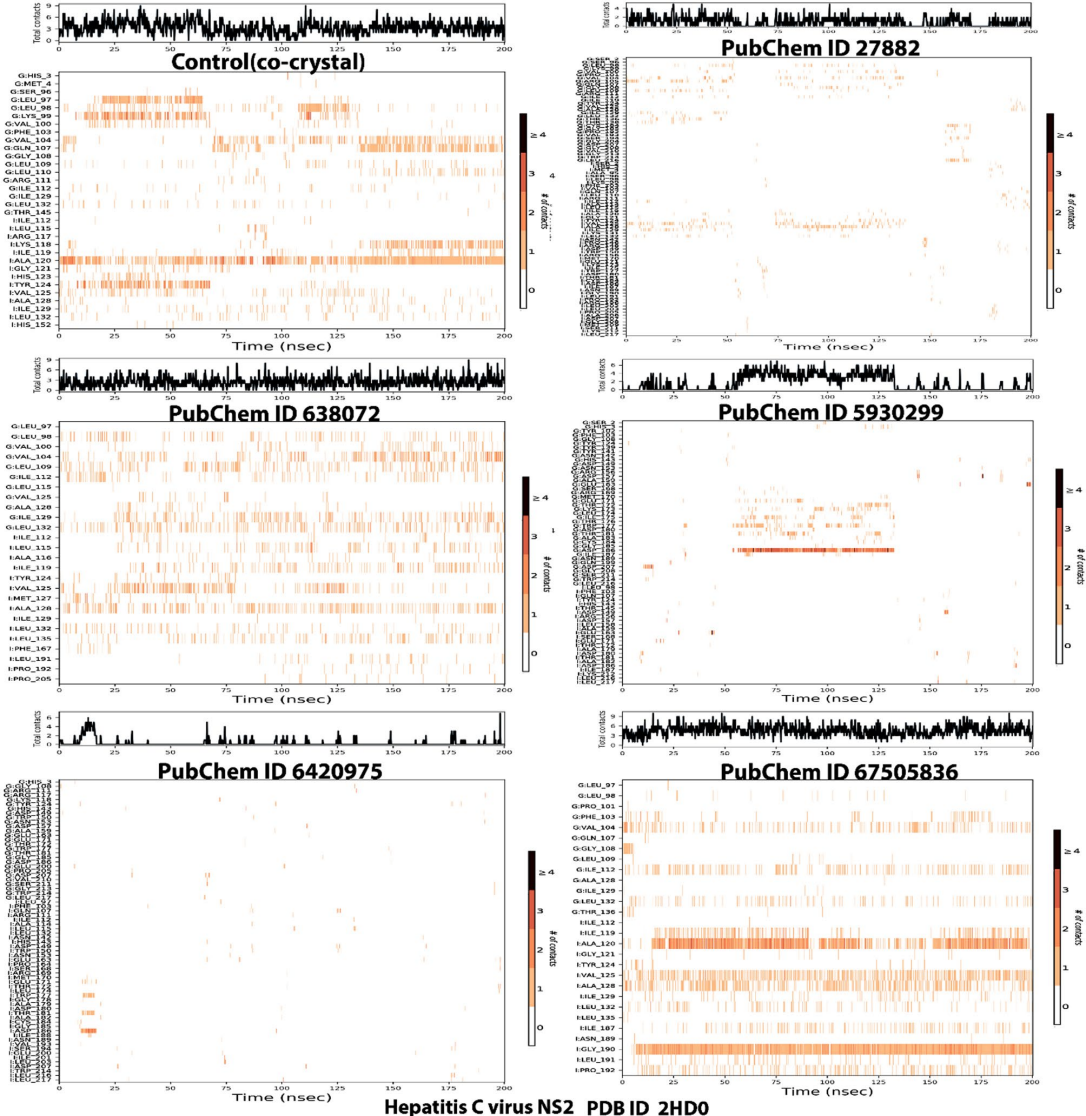
Number of contacts made by the various amino acids during the simulation runs. Key: PubChem CID 27882 (3-Methoxy-4-methylaniline); PubChem CID 638072 (squalene); PubChem CID 5930299 (2,2’-Azopyridine); PubChem CID 6420975 (Isopropyl thiophosphondiamide) and PubChem CID 67505836 (Ledipasvir).

**Fig. 8:**
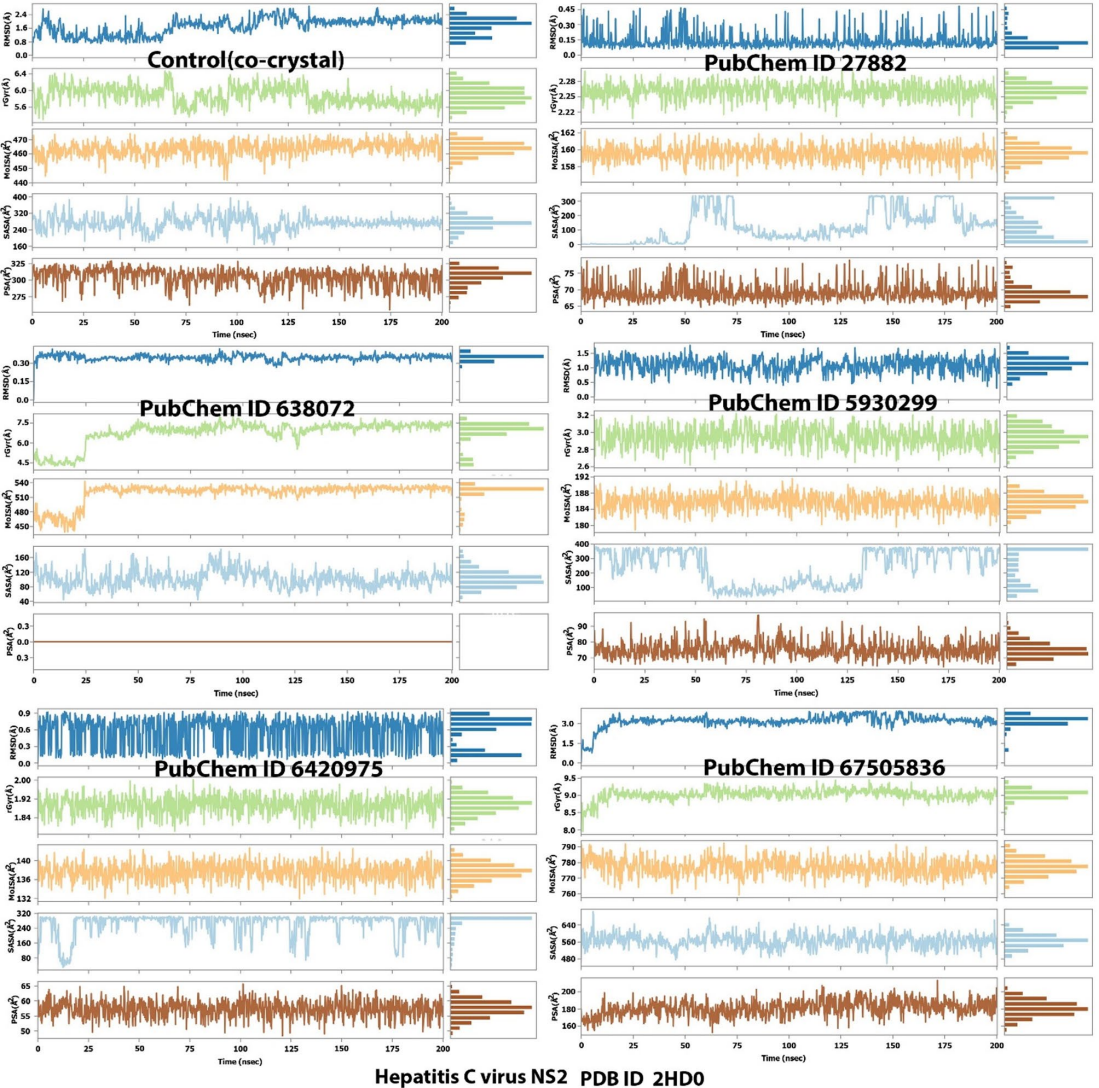
Ligand interaction properties (RMSD, rGyr, MolSA, SASA and PSA). Key: PubChem CID 27882 (3-Methoxy-4-methylaniline); PubChem CID 638072 (squalene); PubChem CID 5930299 (2,2’-Azopyridine); PubChem CID 642075 (Isopropyl thiophosphondiamide) and PubChem CID 67505836 (Ledipasvir).

In addition to the MD, MDS was also conducted (Figs. 4, 5, 6, 7 and 8). RMSD was done elucidate the thermodynamics and stability of the formed complexes during the docking due to the inability of docking to reveal this^30,31^. The MDS results are presented in Figs. 4, 5, 6, 7 and 8. Figure 4 illustrates the RMSD fluctuations during the simulation run for all the complexes. MSD as a parameter is used to evaluate the stability of a protein. An RMSD value that is close to zero indicates perfect resemblance to a control complex. In practical terms, it is impossible for a value of zero to be attained^31^. In practice, RMSD values less than or equals to 2.5 Å and less indicate stability. On the other hand, values greater than 2.5 Å indicate instability. All the formed complexes returned RMSD values that were generally higher than 2 Å, indicating instability. However, the complex between isopropyl thiophosphondiamide and protein revealed RMSD for the ligand that went below 2 Å at various intervals during the simulation runs. The regions that returned stable RMSD values correspond to the amino acid residues that dominate the catalytic region of the target protein.

On the other hand, RMSF as a parameter measures the flexibility and motion of the amino acid residues during the simulation duration. Like RMSD, RMSF values less than 2 Å for a protein indicate resemblance to one another. Figure 5 shows the RMSF fluctuations for the various ligands during the 200 nsec simulation runs. The result indicates fluctuations at various amino acid residues; however, the RMSF values were between 1 and 6 Å. For the control co-crystal, the RMSF values for the ligand was generally below 4.0Å at residues 0-110 and 150–210 but spikes above 4.0Å were observed at residues 25–50 and 125–130. For 3-Methoxy-4-methylaniline, spikes above 6Å, were observed at residues 0–40, 80 and 250, while for squalene, spikes above 6Å were also observed at residues 30, 100–135 and 200–250. For 2,2’-Azopyridine, spike above 6Å were observed at residue positions 80–120 and 210. For isopropyl thiophosphondiamide, the RMSF values were stable but spikes above 6Å were observed at residues 80 and 120, while for ledipasvir, the RMSF values were all below 4.8Å, making it the most stable. Overall, squalene and 2,2’-Azopyridine were most unstable as their RMSF values were above 2 Å^30,31^.

Figure 6 depicts the various protein-ligand contacts using bar chart plots of the interactions residues revealing the various bond types involved in the protein-ligand interactions. The amino acid residues involved in the various interactions varied among the ligands. The number of the amino acid residues were 32, 88, 26, 64, 68 and 28, respectively for the control co-crystal, 3-Methoxy-4-methylaniline, squalene, 2,2’-Azopyridine, isopropyl thiophosphondiamide and ledipasvir. The various bonds were hydrogen, hydrophobic, water bridges and ionic bonds and these varied according to the ligands. Hydrophobic bonds was dominant in squalene while the rest of the ligands had hydrogen, hydrophobic, and water bridges as the dominant interactions. The presence of water bridges is important as they can act as donors as well as acceptors of electrons, and also aid maintain stability in the vicinity of the active site^50^. The presence of hydrophobic bonds have been shown to facilitate the stability of the ligands when bound to the active site of the protein^51^. The combination of hydrophobic and hydrogen bonds contributes to the overall stability of the ligand-target protein complex that is formed^52^.

Figure 7 shows the frequency of contacts made by the various resides during the simulation runs for the various ligands. The different color bands on each of the contact plot for each ligand indicates the number of contacts made by the various ligands. The result indicates that the control co-crystal had the higher number of amino acids that made contacts (1 to 3) with the ligand throughout the simulation (Tyr124, Ala120, Gln104, Gln17, Lys99, Leu98, and Leu 97) while ligands 3-Methoxy-4-methylaniline and Isopropyl thiophosphondiamide had the least contract (0–1). The ligand squalene had as many contacts as the control co-crystal but the contacts were less strong compared to the control co-crystal. Similarly, the control drug had as many contacts as squalene and the control co-crystal with Gly 190 and Ala 120 making contacts almost throughout the simulation duration. The ligand 2,2’-Azopyridine made a few contacts with Asp 106 made the strongest contact around 50 to 125 nsec compared to the rest of the amino acids.

To further reveal the stability of the ligands and their drug-likeness properties, ligand properties were evaluated and these includes RMSD, rGyr, MolSA, SASA and PSA. For ligand Isopropyl thiophosphondiamide, its RMSD value was stable throughout the duration of the simulation with values that ranged from 0.15 to 0.90 Å. Ligand 638073 gave unstable RMSD value throughout the 200 nsec. On the other hand, 2,2’-Azopyridine and 3-Methoxy-4-methylaniline gave stable RMSD value throughout the simulation run. The control co-crystal gave RMSD values that were only stable during the first 60 ns but slight unstable afterwards. The radius of gyrations values varied for the various ligands. The ranges of the values were 5.6 to 6.0, 2.22 to 2.28, 4.5 to 7.5, 2.6 to 3.2, 1.84 to 2.00 and 8.5 to 9.5 Å for the control (co-crystal), ligands 3-Methoxy-4-methylaniline, squalene 2,2’-Azopyridine), isopropyl thiophosphondiamide and ledipasvir, respectively. The closeness of the values of the radii of gyration of the test ligands indicates the compactness of the complexes formed^52^. The MolSA, SASA and PSA values also varied according to the various ligands. The larger the values of these parameters (MolSA, SASA and PSA), the more of the complex that is available for contact with water and also indicates stability^53^.

Put together, all the test compounds utilised in this study show favourable docking, and simulation properties. However, it is important to have an understanding of their chemistry. Squalene is a natural triterpene whose chemistry is well known, as well as its wide range of antimicrobial (antiviral and anticancer) activities^54^. Its mode of action includes its ability to target squalene synthase, a key enzyme in cholesterol biosynthesis, and in the process reduce HCV replication and assembly^55,56^. As indicated by the highest docking scores by squalene and the abundance of diverse bonds (hydrogen, ionic, and hydrophobic) formed with various amino acids with the active site of the HCV protease. Thus, it is possible that squalene may interfere with the activity of the target via its interference with polyprotein cleavage and replication. As a compound, 2,2’-Azoxybis [3-methylpyridine] contains an azoxy functional group and pyridine rings that have the potential to interact with the target via the formation of hydrogen bonds or π-π stacking interactions with key residues in the active site^57^. This aligns with the docking scores obtained in our study and the diverse interactions of this compound with the amino acid residues in the active site of the protease. Furthermore, a study revealed pyridine, one of the key components of 2,2’-Azoxybis [3-methylpyridine], to have antimicrobial and antiviral properties but not against HCV. By interacting with diverse amino acids within the active site of the protein, 2,2’-Azoxybis [3-methylpyridine] can interfere with the assembly and release function of the protease complex^13,14^.

On the other hand, isopropyl thiophosphonodiamide is an organophosphorus compound that is well known for its ability to act as an inhibitor of proteases, forming covalent or non-covalent interactions with catalytic residues^58^. Our docking scores indicate that isopropyl thiophosphonodiamide was able to bind to the target protein via diverse bond types. There is no reported activity for isopropyl thiophosphonodiamide. However, it has been reported that thiophosphonates can mimic natural protease substrates and, the process, potentially bind irreversibly to the active protease^58^. Furthermore, it has been shown that synthesised phosphonate and thiophosphonate peptide analogues could be potential peptide inhibitors of proteases^59^. It has also been reported that a phosphonate inhibitor inhibited the NS2B/NS3 protease of the West Nile virus^60^. Thus, it is possible that isopropyl thiophosphonodiamide could interfere with the function of the protease in this study by acting as a pseudo substrate for the protease. Aromatic amines like 3-methoxy-4-methylaniline possess methoxy and methyl groups whose medicinal chemistry properties are well known^61^. Furthermore, it has been shown that the methoxy and methyl groups have the capacity to enhance lipophilicity and allow better penetration into the protease binding pocket and increase bioavailability^62^. However, these have not been reported for HCV. In this study, 3-methoxy-4-methylaniline interacted with the protease using diverse bond types even though it returned the least docking score. Thus, it is possible that it could exert its effect by interfering with the protease by decreasing its assembly and release function following enhanced bioavailability.

## Conclusion

The ligands 3-methoxy-4-methylaniline, 2,2’-azoxybis[3-methylpyridine], isopropyl thiophosphondiamide, and squalene identified in *J. tanjorensis* and *S. nigrum* plant species showed not toxicity. The compounds further showed drug-like properties by obeying the Lipinski rule. The HOMO-LUMO and global reactivity descriptors showed mobility of electrons between the frontier molecular orbitals and the entire compounds, dictating the compounds to be moderately stable and highly reactive. Docking scores of the ligands ranged from − 3.5 to -7.3 kcal/mol and was slightly lower than the standard drug, ledispasvir (-8.8 kcal/mol). Various bond types were utilised in the docking against the target protein further affirming their ability to potentially interfere with the activity of the target protease. The amino acid residues involved in the docking were all those within the active site of the target protein. Radius of gyration values suggest that the ligands can form complexes that are compact. The RMSD and RMSF values suggested the ligands were slightly stable while the MolSA, SASA and PSA indicate that they have adequate surface areas for water contact and are stable. The selected compounds offer a ray of optimism for addressing hepatitis C virus-related infections and diseases via diverse mechanisms; however, further in vitro and in vivo studies are required to validate these favourable properties observed in this study.

### Limitation of the study

Our finding indicates that bioactive compounds from medicinal plants used by locals to manage HCV infection possess anti-HCV properties. However, some limitations abound. First, our study design was based on an in-silico approach. Hence the findings have to be validated using in vivo and in vitro studies. Secondly, our simulation, though kept at 200 nsec, was still able to capture the stability of the formed complexes. Thirdly, we did not evaluate all the bioactive compounds from both plants but only utilised those with the highest abundance in terms of peaks.

## Data Availability

Data is provided within the manuscript.
